# High throughput exome coverage of clinically relevant cardiac genes

**DOI:** 10.1186/s12920-014-0067-8

**Published:** 2014-12-11

**Authors:** Dorin Manase, Lisa CA D’Alessandro, Ashok Kumar Manickaraj, Saeed Al Turki, Matthew E Hurles, Seema Mital

**Affiliations:** Division of Cardiology, Department of Pediatrics, Hospital for Sick Children, University of Toronto, Toronto, Ontario Canada; Program in Genetics and Genome Biology, The Hospital for Sick Children, Toronto, Ontario Canada; Wellcome Trust Sanger Institute, Hinxton, UK

**Keywords:** Exome sequencing, Coverage, Congenital heart disease, Cardiac, Genomics

## Abstract

**Background:**

Given the growing use of whole-exome sequencing (WES) for clinical diagnostics of complex human disorders, we evaluated coverage of clinically relevant cardiac genes on WES and factors influencing uniformity and depth of coverage of exonic regions.

**Methods:**

Two hundred and thirteen human DNA samples were exome sequenced via Illumina HiSeq using different versions of the Agilent SureSelect capture kit. 50 cardiac genes were further analyzed including 31 genes from the American College of Medical Genetics (ACMG) list for reporting of incidental findings and 19 genes associated with congenital heart disease for which clinical testing is available. Gene coordinates were obtained from two databases, CCDS and Known Gene and compared. Read depth for each region was extracted from the exomes and used to assess capture variability between kits for individual genes, and for overall coverage. GC content, gene size, and inter-sample variability were also tested as potential contributors to variability in gene coverage.

**Results:**

All versions of capture kits (designed based on Consensus coding sequence) included only 55% of known genomic regions for the cardiac genes. Although newer versions of each Agilent kit showed improvement in capture of CCDS regions to 99%, only 64% of Known Gene regions were captured even with newer capture kits. There was considerable variability in coverage of the cardiac genes. 10 of the 50 genes including 6 on the ACMG list had less than the optimal coverage of 30X. Within each gene, only 32 of the 50 genes had the majority of their bases covered at an interquartile range ≥30X. Heterogeneity in gene coverage was modestly associated with gene size and significantly associated with GC content.

**Conclusions:**

Despite improvement in overall coverage across the exome with newer capture kit versions and higher sequencing depths, only 50% of known genomic regions of clinical cardiac genes are targeted and individual gene coverage is non-uniform. This may contribute to a bias with greater attribution of disease causation to mutations in well-represented and well-covered genes. Improvements in WES technology are needed before widespread clinical application.

**Electronic supplementary material:**

The online version of this article (doi:10.1186/s12920-014-0067-8) contains supplementary material, which is available to authorized users.

## Background

Next generation sequencing (NGS) has changed the diagnostic landscape for complex human disorders. Sequencing costs, particularly for exome sequencing, have plummeted over the last decade [1,2] allowing for the rapid uptake of technology in both the research and clinical realms. NGS platforms capitalize on parallel processing to generate hundreds of megabases of sequence from a single run allowing for deep coverage of vast regions [3]. A greater understanding of the limitations and potential pitfalls of sequencing technology is essential before clinical uptake of this technology [4]. Whole exome sequencing (WES) represents one NGS platform that has been effective in the study of rare Mendelian diseases and has become widely popular as an efficient method of identifying genetic variants within an individual’s genes. Currently WES has two major limitations: lower accuracy in nucleotide calls compared with Sanger sequencing and the lack of uniform coverage of exonic regions. The considerably higher rate when compared to Sanger sequencing of both false-negative and false-positive calls, can be minimized by increasing the depth of sequencing [5]. However, variant identification in whole exome sequencing is still limited by the non-uniformity of overall coverage which in turn is determined by sequence capture and read depth. Previous studies report considerable variability in uniformity and depth of coverage across the exome including base-to-base and gene-to-gene coverage [6-9]. Although newer versions of capture kits are designed to target a greater number of bases, it is unclear if capture of exonic loci is uniform across genes. In 2013, the American College of Medical Genetics (ACMG) published recommendations for reporting of incidental findings in clinical exome sequencing. Since a large number of genes for cardiac disorders are included in the ACMG reporting list [10], we assessed targeted capture and coverage of clinically relevant cardiac genes with whole exome sequencing and investigated factors contributing to heterogeneity in coverage.

## Methods

### Whole exome sequencing and candidate gene selection

Participants from six centers in Ontario were recruited prospectively to the Heart Centre Biobank Registry at the Hospital for Sick Children in Toronto. Details of the Biobank Registry have been published previously [11]. All probands/parents/legal guardians provided informed consent and the study was approved by the local research ethics boards at all participating sites (Hospital for Sick Children, Hamilton Health Sciences Centre, London Health Sciences Centre, Kingston General Hospital, Children’s Hospital of Eastern Ontario, and Toronto General Hospital). Probands with congenital heart disease and affected and unaffected relatives who underwent whole exome sequencing were included. All sequencing was performed using Illumina HiSeq with sequence capture performed using 3 different versions of the Agilent SureSelect Human All Exon capture kit. Twenty individuals were sequenced using Agilent 44 MB Version 2 (V2), 94 individuals were sequenced using the 50 MB Version 3 (V3), and 93 individuals were sequenced using the 51 MB Version 4 kit (V4). Additionally, we analyzed 6 exomes that were sequenced using the 50 MB Version 5 kit (V5) provided by the Wellcome Trust Sanger Institute. All exomes were aligned to the human genome version 19 using Burrows-Wheeler Aligner [12] to generate BAM files, and variant calling was performed using the Genome Analysis Tool Kit (GATK) [13]. For the purpose of coverage analysis, 50 clinically relevant cardiac genes were selected. These included 31 genes associated with cardiac disorders derived from the ACMG list for reporting of incidental findings i.e. genes associated with congenital heart disease (CHD), cardiomyopathies, vascular and rhythm disorders [10], and 19 additional genes associated with CHD not on the ACMG reporting list for which sequencing-based clinical genetic testing is currently available (Table 1).

**Table 1:** **Clinically relevant cardiac gene list**

	**Phenotype**	**Associated genes**
**American College of Medical Genetics**	▪ Hypertrophic cardiomyopathy	*MYBPC3, MYH7, TNNT2, TNNI3, TPM1, MYL3, ACTC1, PRKAG2, GLA, MYL2, LMNA*
	▪ Dilated cardiomyopathy	
	▪ Catecholaminergic polymorphic ventricular tachycardia	*RYR2*
	▪ Arrhythmogenic right ventricular cardiomyopathy	*PKP2, DSP, DSC2, TMEM43, DSG2*
	▪ Romano–Ward long QT syndrome	*KCNQ1, KCNH2, SCN5A*
	▪ Brugada syndrome	
	▪ Familial hypercholesterolemia	*LDLR, APOB, PCSK9*
	▪ Ehlers–Danlos syndrome, vascular type	*COL3A1*
	▪ Marfan syndrome	*FBN1, TGFBR1, TGFBR2, SMAD3, ACTA2, MYLK, MYH11*
	▪ Loeys–Dietz syndromes	
	▪ Familial thoracic aortic aneurysms and dissections	
**CHD genes**	▪ Genes associated with congenital heart disease	*BRAF, NOTCH1, CFC1, NRAS, CHD7, PTPN11, GATA4, RAF1, HRAS, SOS1, JAG1, TBX1, KRAS, TBX5, MAP2K1, ZIC3, ELN, NKX2-5, NF1*

Gene list includes American College of Medical Genetics list for reporting of incidental findings (n = 31) and genes associated with congenital heart disease (CHD) for which clinical testing is available (n = 19). All genes are listed according symbol.to their HUGO Gene Nomenclature Committee (HGNC).

### Exonic regions represented in reference datasets

Agilent capture kits are primarily designed to target genes based on the National Center for Biotechnology Information Consensus Coding Sequence (CCDS) dataset. The CCDS database [14] is built by consensus among four major collaborating partners: European Bioinformatics Institute, National Center for Biotechnology Information (NCBI), Wellcome Trust Sanger Institute, and the UCSC [15-17]. The University of California, Santa Cruz (UCSC) Known Gene dataset houses a larger database that encompasses not only all the CCDS transcripts but also any protein coding genes that are substantiated by a transcript in GenBank mRNA or NCBI RefSeq and have a UniProt protein. Alternative splicing isoforms are also included in Known Gene if they represent a UniProt protein and have a transcript [18]. We therefore compared how well each gene was represented in both datasets by first obtaining gene coordinates for all protein coding regions of every available transcript in BED format from the CCDS and the Known Gene databases using the UCSC table browser [19]. BEDTools [20] was then used to collapse coordinates to unique locations in order to avoid overlap and also ensure all CCDS coordinates were contained within the Known Gene coordinates. For each gene the exonic regions included within the CCDS database were represented as a percentage of the Known Gene superset for that gene.

### Cardiac gene coverage

Coverage across the 50 cardiac genes was analyzed as a combination of sequence capture and read depth. The read depth at each nucleotide was extracted from each subject’s exome BAM file using SAMtools *mpileup* [21]. Subsequent data handling was performed with custom scripting using the Python programming language (v2.7.1) [22]. The read depth or coverage was the number of independent times a particular nucleotide is represented in a collection of raw sequences, and was expressed numerically as 1X, 2X, 30X, etc. [23]. The read depth in every captured location was averaged across the samples sequenced for each capture kit. This produced an average capture per coordinate within both the CCDS and Known Gene datasets for each kit that was used in subsequent analyses.

### Gene size and GC content

Gene size was calculated as the sum of unique bases of each candidate cardiac gene from the CCDS dataset. GC content was analyzed using BEDTools’ *nuc* function, along with UCSC’s human genome version 19 FASTA file, to output counts for each of the four nucleotides in a given region.

### Statistical analysis

The proportion of bases in Known Gene regions captured by different versions of CCDS-based capture kits was compared. The comparison was performed across the entire targeted region as well as a gene by gene comparison to identify which genes had the highest discordance between databases. Actual coverage of the targeted region at a minimum read depth of 3X (the minimum depth required to identify a heterozygous mutation) and at an optimal read depth of 30X or higher (the minimum requirement for a confident genotype call) was then determined and influence of capture kit version and observed sequencing depth on coverage was analyzed. Uniformity of coverage was assessed across the candidate genes by calculating the median coverage of each gene as well as the variability in base to base coverage within each gene. Inter-sample variability was assessed for all bases within the CCDS targets by plotting the read depth confidence interval across all samples. Finally, the association between gene size and GC content on coverage of candidate cardiac genes was analyzed using linear regression. All statistical analysis was performed using the R statistical programming package (v3.0.1) [24].

## Results

### Study cohort

207 participants underwent whole exome sequencing between April 2012 and January 2013. 196 participants had a diagnosis of CHD and 17 had no structural heart defects. The average age at enrollment was 14.7 ± 11.7 years. 109 participants (53%) were male. Racial distribution included 85% Caucasians, 9% Asians, 1% African American, and 5% of mixed or unknown ethnicity. Six additional exome datasets were obtained from the Wellcome Trust Sanger Institute HapMap data with no information on disease status available.

### Cardiac gene sequences represented in CCDS vs. known gene

The Agilent capture kits are designed to capture sequence data derived from the CCDS dataset. For the 50 cardiac genes, this amounts to a total of 166,271 bp of unique protein coding bases. For the Known Gene dataset, this amounts to 300,468 bp unique protein coding bases. Additional file 1: Table S1 shows the proportion of Known Gene regions that are also included in the CCDS. Overall only 55% of the regions within the Known Gene dataset for all genes were represented in the CCDS dataset. At individual gene level, this ranged from 10.1% (*KRAS*) to 96.8% (*APOB*).

### Capture of cardiac genes by kit version

**Figure 1:**
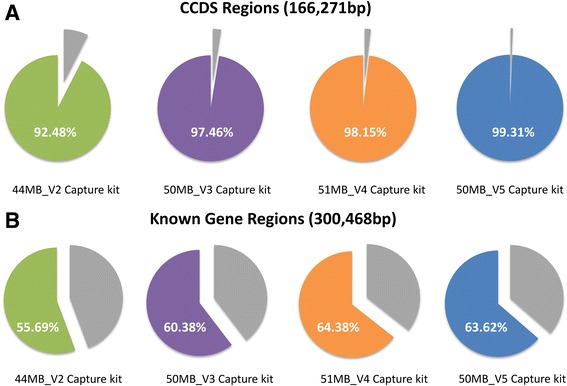
**Agilent capture kit target size.** Graphical representation of the proportion of bases captured by the Agilent 44MB_V2 (green), 50MB_V3 (purple), 51MB_V4 (orange), and 50MB_V5 (blue) capture kits. The grey wedges indicate the proportion not captured by the kits. **A**. Percentage of the total number of bases from CCDS genomic locations for all 50 clinically relevant cardiac genes. **B**. Percentage of the total number Known Gene genomic locations for all 50 clinically relevant cardiac genes.

The extent to which each capture kit targeted the candidate gene sequences of the 50 cardiac genes was first assessed (Figure 1). For research studies, exomes are currently sequenced between 30X to 50X depth of coverage to confidently distinguish heterozygous or homozygous mutations from misaligned reads or artifact. However, to compare overall capture between various capture kits, regions covered at a read depth of at least 3X were included. At a read depth of 3X or higher, the Agilent V2 captured 92% of the CCDS cardiac gene region. This increased to 97% with Agilent V3, 98% with Agilent V4, and 99% with Agilent V5. While each newer kit version showed improved capture of CCDS regions, capture of the Known Gene regions remained low. The Agilent V2 captured only 55% of the Known Gene cardiac regions. Captured improved to 60% with V3, 64% with V4, and 63% with V5. The exonic regions within the CCDS versus Known Gene database targeted and captured for each gene are shown in Additional file 1: Table S1. Only 55-64% of the Known Gene regions were captured by the current CCDS-based capture kits. Therefore, although newer capture kit versions showed improvement in the amount of CCDS regions captured reaching up to 99%, the Known Gene capture only improved marginally up to 64% with the V5 kit showing no improvement over its predecessor V4.

### Uniformity of coverage of cardiac genes

**Figure 2:**
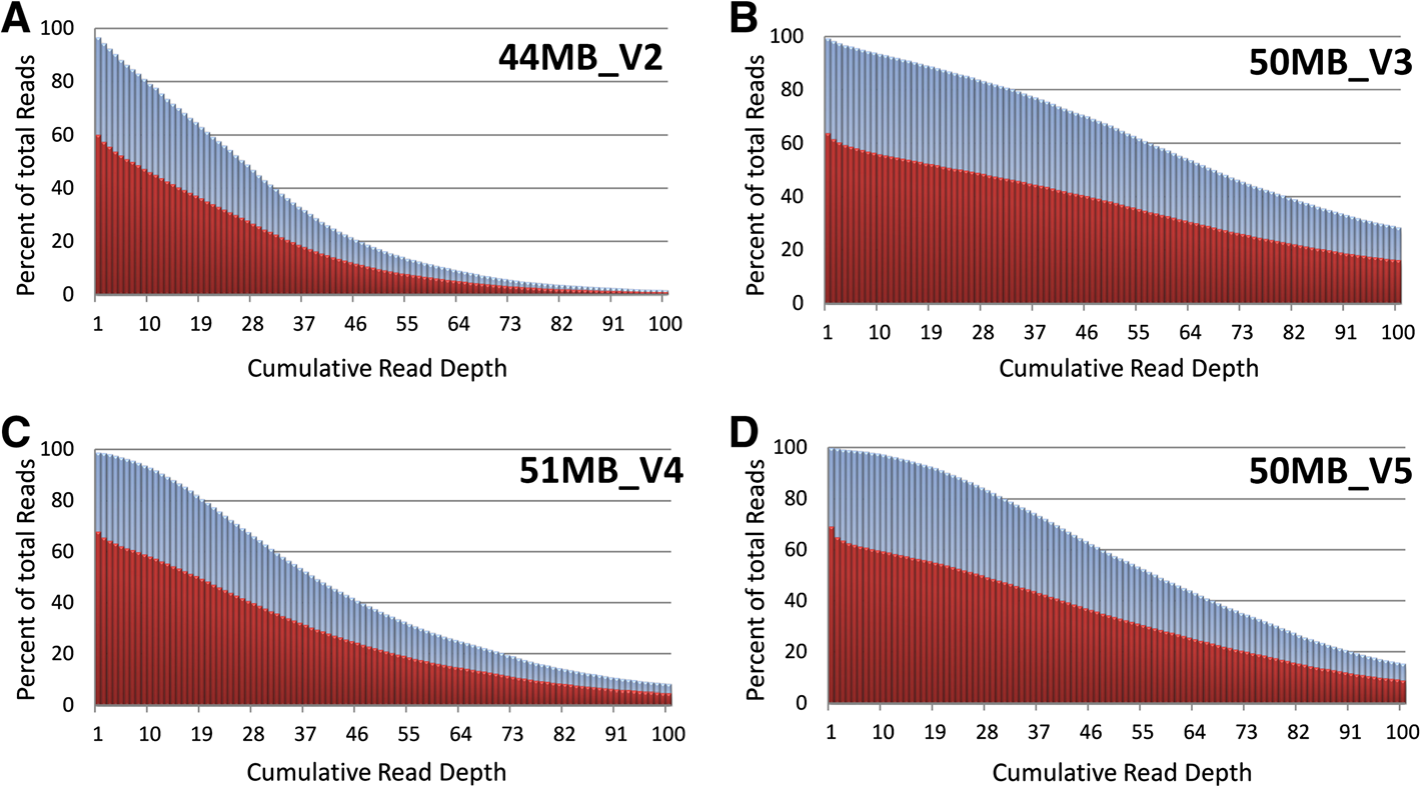
**Cumulative coverage with Agilent capture kit versions.** Graph showing proportion of CCDS and Known Gene datasets covered at various read depths by four Agilent capture kit versions. Coverage of CCDS coordinates is shown in blue while coverage of Known Gene coordinates is depicted in red. There was good coverage, 92-99% of CCDS target regions, at a minimum read depth of 3X but only 55-64% coverage of Known Gene target regions at 3X. **A**. Proportion of CCDS and Known Gene read depths for the Agilent 44MB_V2 capture kit. **B**. Proportion of CCDS and Known Gene read depths for the Agilent 50MB_V3 kit. This capture kit version demonstrated the highest overall observed read depth. **C**. Proportion of CCDS and Known Gene read depths for the Agilent 51MB_V4 kit. **D**. Proportion of CCDS and Known Gene read depths for the Agilent 50MB_V5 kit.

**Figure 3:**
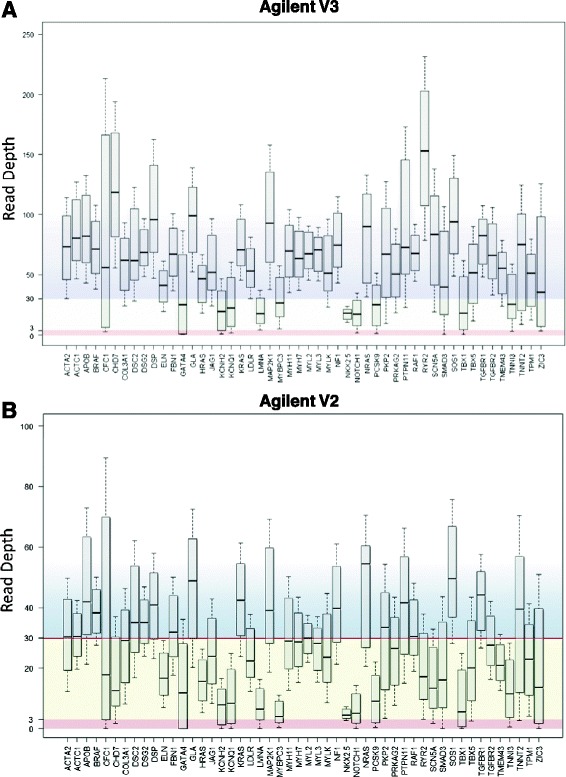
**Gene by gene coverage of cardiac genes. A**. Box-and-whisker plots showing median read depth and 25% to 75% interquartile ranges (IQR) for the 50 cardiac genes in 94 exome samples captured using Agilent V3 that had high observed sequencing depth, 80X. Plots represent the read depth coverage for transcripts found within the CCDS. There was variability in depth of coverage between genes and variable coverage within genes as shown by the wide IQR. Based on the CCDS dataset, 10 of the 50 genes had median read depth below 30X and only 32 genes had an IQR above 30X. **B**. Box-and-whisker plots showing median read depth and 25% to 75% interquartile ranges (IQR) for the 50 cardiac genes in 20 exome samples captured using Agilent V2 that had the lowest observed sequencing depth, 30.5X. Based on the CCDS dataset, 30 of the 50 genes had median read depth below 30X and only 5 genes had an IQR above 30X.

**Figure 4:**
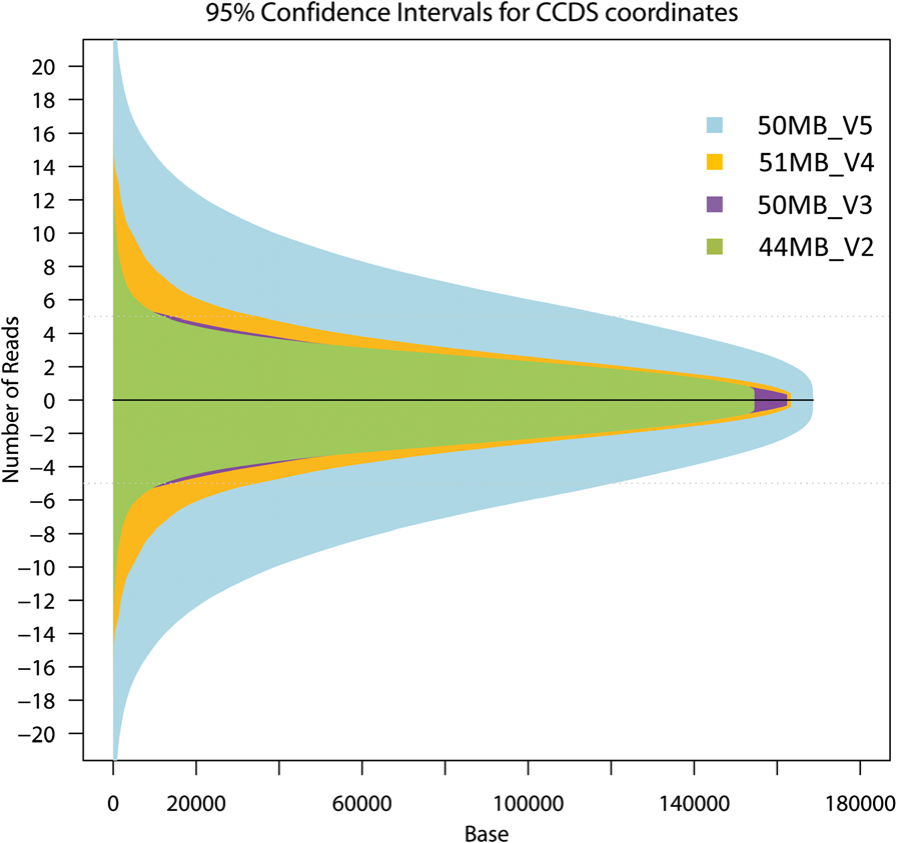
**Inter-sample variability of observed read depths.** Plot of the 95% confidence interval per captured CCDS base across samples for the Agilent 44MB_V2 (green) 50MB_V3 (purple), 51MB_V4 (orange), and 50MB_V5 (blue) capture kits. For V2, V3, and V4 kit versions, over 86% of captured bases varied less than ±5 reads between samples, with the largest variation coming at higher read depths. Given that V5 had only six exomes, the variability is more pronounced due to the small sample size, however, over 85% of captured bases varied by more than ±10 reads between samples. The largest variability was seen at higher read depths (>30X). The depth of sequencing and version of the capture kit did not affect inter-sample variability.

Since each capture kit was sequenced at a different intended depth, we used the exome dataset that had the highest observed average read depth for further analysis of uniformity of coverage across cardiac genes. The observed average read depth for Agilent V2 capture kit was 30.5X, for the Agilent V3 capture kit was 80X, for the Agilent V4 capture kit was 48X, and for the Agilent V5 capture kit was 63X (Figure 2). Therefore, given that the Agilent V3 capture kit had the highest overall observed read depth, this dataset (n = 94) was further analyzed to assess the uniformity of coverage of each of the 50 cardiac genes. Despite high read depths, Figure 3A shows the considerable variability in coverage between genes and within genes. Ten genes were covered at a median read depth of less than the optimal 30X. These included 2 genes associated with long QT syndrome (types 1, 2, and 3) and Brugada syndrome (*KCNQ1, KCNH2*), 3 genes associated with hypertrophic and dilated cardiomyopathy (*LMNA, MYBPC3, TNNI3*), 1 gene associated with familial hypercholesterolemia (*PCSK9*), and 4 genes associated with CHD (*GATA4, NKX2-5, NOTCH1, TBX1*). For comparison Figure 3B shows the same plot but for the Agilent V2 data that had the lowest observed average read depth of 30.5X. Here we see that 30 of the 50 genes had median read depth below 30X and only 5 genes had an IQR above 30X. Expressing gene coverage as a median read depth does not imply that all bases within that gene are covered at the same depth. A small number of highly covered bases may skew the average toward the higher end despite the majority of bases having low read depth. Therefore the inter-quartile range (IQR) for base-by-base coverage for each gene was measured. Only 32 of the 50 genes examined had the majority of their bases covered at an IQR ≥30X. To determine if there was inter-sample variability, the 95% confidence interval per captured CCDS base by each of the three capture kits across samples was calculated (Figure 4). Despite the use of different capture kits and different intended sequencing depths, the consistency in capture across exomes was maintained with very minimal inter-sample variability (over 85% of bases fell within ±5 reads, with the exception of the V5 capture kit data that only included 6 exomes and therefore 85% of bases fell within ±10 reads). Overall, these findings suggest that while higher sequencing depths improved average coverage across targeted genomic regions, the gene-by-gene and base-by-base coverage remained non-uniform.

### Association of gene size and GC content with genomic coverage

**Figure 5:**
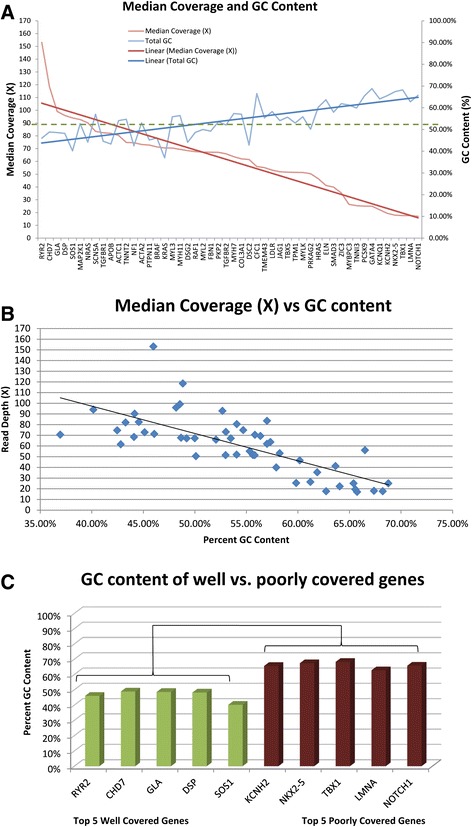
**GC content analysis. A**. GC content (blue) and median coverage (red) for each of the 50 cardiac genes was plotted in decreasing order of from highest to lowest coverage. The linear average was plotted as the dark line in blue for GC content and red for median coverage. These linear averages show that as median coverage decreased from left to right, GC content increased. **B**. For each of the 50 genes, the median coverage and GC content percentage was plotted. There was a negative correlation between median coverage and total GC content of the 50 cardiac genes (r^2^ = 0.547; p = 8.66 × 10^−10^). **C**. Percent GC content of the top five well covered genes in green and the bottom five poorly covered genes in red showed a significant difference (p = 1.48 × 10^−5^).

The potential contribution of gene size and GC content to the gene-to-gene variability in coverage was evaluated. Gene size showed a significant but modest correlation with median coverage (r^2^ = 0.19; p = 1.46 × 10^−3^) and accounted for less than 20% of the variance in coverage of the cardiac genes. There was however a strong negative correlation between mean coverage and GC content as higher GC content was associated with lower median coverage per gene (Figure 5A). Figure 5A shows median coverage and GC content of each gene sorted with highest coverage on the left. Figure 5B shows the significant inverse relationship between GC content and median coverage (r^2^ = 0.55; p = 8.66 × 10^−10^). Figure 5C shows the significant difference in percent GC content of the 5 best-covered genes and the 5 worst-covered genes (p = 1.48 × 10^−5^). In fact, of the 50 cardiac genes, genes with highest coverage had a GC content of <50% while poorly covered genes had a GC content of >50%.

## Discussion

The increasing use of next generation sequencing for research and clinical genetics and the move towards scanning the genome or exome not only for variation in targeted genes of relevance to the disease under study, but also for variant-detection in other disease-associated genes has intensified the focus on the technical accuracy and validity of the technology [10]. Coverage analysis of 50 clinically important cardiac genes using data from research based WES demonstrated considerable base-by-base and gene-by-gene variability in coverage within and across the candidate genes. Sources of variability included incomplete targeting of exonic regions, low capture efficiency, low sequencing depth, larger gene size and high GC content within the target region. Despite improvement in median coverage across the exome with newer capture kits and with higher sequencing depths, the coverage of individual genes was non-uniform. Overall, these results highlight the importance of assessing adequacy of coverage of target genes when analyzing whole exome data for disease genes and the need to improve current technology before whole exome sequencing can be widely deployed towards clinical cardiac diagnostics.

The first important finding was incomplete capture of clinically important cardiac genes with whole exome sequencing. Currently, exome sequencing capture kits are primarily designed to capture regions identified within the CCDS database. However, unlike the Known Gene database, the CCDS does not contain all possible transcripts, thereby restricting target regions to just over 50% of known genomic regions. Missing exonic nucleotides not captured with these kits may result in non-detection of variants in alternate isoforms. The difference in exonic region definition varies greatly at the gene level as shown in Additional file 1: Table S1 where the number of bases per gene for the 50 cardiac genes in the CCDS dataset can range from 10% to 96% of their size in the Known Gene dataset. Given that some known transcripts identified within the Known Gene dataset have yet to be approved in the CCDS dataset, there is a potential bias in gene discovery towards genes that are already well defined within the CCDS. Further increase in target regions is necessary to enhance more uniform and complete capture across the exome.

The most important metric of efficiency for a capture experiment is the proportion of targeted DNA inserts that are specifically hybridized and recovered from the capture. Our results showed that, for the 50 genes studied, minimum coverage (3X) was obtained to a high degree (92% to 98%) for all the capture kit versions within the CCDS. Higher depth of sequencing can improve coverage across difficult targets and as expected, the proportion of the genomic regions with high read depth was improved when intended sequencing depth was also higher with optimal coverage (30X) reached for 82.0% of the targets that were sequenced using the Agilent V3 kit that had an observed average depth of 80X. Despite overall improvement in coverage, there remained significant gene-to-gene variability in coverage. Ten of the 50 cardiac genes (including 6 on the ACMG list) had a median coverage that was less than optimal (<30X), increasing the risk of under-detection of true positives (Type II error). Thus a high intended depth of sequencing can improve the efficiency of capture but it increases costs and did not increase the number of genomic regions covered. Improving coverage will therefore require improvement in the capture technology and probe capture design to capture previously missing regions of the exome.

Gene-to-gene variability in coverage was not accounted for by inter-sample variability, which was consistently low across all 50 genes, and only modestly related to the size of the exonic regions examined for each gene. It did however correlate with GC content with lower coverage in regions that showed a high GC content. This limitation in sequencing regions with high GC content has been identified previously [6,25]. Targeted re-sequencing and modified sequencing techniques, such as oligonucleotide-selective sequencing, may provide a more efficient strategy to deal with GC rich genes [26]. Therefore, a preliminary screen for the amount GC content of candidate genes prior to sequencing is recommended.

In summary, these results highlight the importance of assessing adequacy of coverage of candidate or target genes in genomic analyses to avoid false negative results. When comparing cohorts sequenced using different capture kits or sequencing platforms, coverage analysis plays an even more significant role. Underreporting of variants due to lower coverage in one cohort can result in false ascertainment of enrichment of variants in the well covered cohort resulting in a Type II error. Accurate assessment of coverage between cohorts should also look beyond the commonly reported mean/median depth per base. This type of measure for overall coverage gives no indication of the uniformity of coverage across the gene. Base-by-base read depth comparison should be assessed to ensure cohorts are comparable at specific variant sites.

A limitation of our study is that we did not assess if structural variation, such as common and rare copy number variants (CNVs), contribute to the discrepancy in coverage when analyzing a small subset of genes especially in the presence of population specific CNVs. Another limitation is that we did not study the impact of coverage on variant detection sensitivity. However, several other studies have already evaluated variant detection sensitivity with exome data and identified numerous factors that influence variant detection sensitivity including coverage, quality of alignment and mapping, duplicates, strand bias, and type of algorithm used [5,27-29]. A recent study also found that a mean on-target read depth of 17-37X is required to identify 90% of heterozygous SNVs in the targeted regions, depending on the uniformity of read coverage [30]. Therefore strategies to enhance capture and coverage as well as the sensitivity in variant detection are needed.

## Conclusions

In conclusion, non-uniformity of coverage is a limitation of exome sequencing and may introduce bias into gene and variant discovery. As the application of exome sequencing expands into the clinical domain, careful interpretation of findings in the context of coverage is essential. Improvements to sequencing capture technology to expand the targeted regions and advances in bioinformatic approaches to account for differential coverage are essential to improve clinical implementation. From a research perspective, care should be taken when assessing overall mutation burden or identifying casual variants or genes. There is a bias towards well-covered and easy to sequence genes and a risk of under-reporting of mutation burden in poorly covered genes.

## Additional file

Additional file 1: Table S1. Cardiac genomic regions targeted in the CCDS vs Known Gene datasets. Total size in unique base pairs for all exons of all transcripts contained within the CCDS, Ensembl, and Known Gene datasets for all 50 cardiac genes. The Ensembl database is included for reference as well given it is a widely used data source and therefore provides context for the other databases. The proportion of Known Gene bases represented in CCDS for each gene is shown as percentages as well as the proportion of Ensembl bases represented in CCDS for each gene. The number of bases targeted and captured (minimum 3X) for each capture kit is shown as a percentage of the total bases found in each dataset per gene.

## Abbreviations

ACMG: American College of Medical Genetics
CHD: Congenital heart disease
CCDS: Consensus CDS
CNV: Copy number variant
NGS: Next generation sequencing
WES: Whole exome sequencing.
