# Supplementary material for: High throughput exome coverage of clinically relevant cardiac genes
Source: BMC Med Genomics. 2014 Dec 11;7:67. doi: 10.1186/s12920-014-0067-8 (PMC4272796; doi:10.1186/s12920-014-0067-8)
Supplement: Additional file 1: Table S1. — Cardiac genomic regions targeted in the CCDS vs Known Gene datasets. Total size in unique base pairs for all exons of all transcripts contained within the CCDS, Ensembl, and Known Gene datasets for all 50 cardiac genes. The Ensembl database is included for reference as well given it is a widely used data source and therefore provides context for the other databases. The proportion of Known Gene bases represented in CCDS for each gene is shown as percentages as well as the proportion of Ensembl bases represented in CCDS for each gene. The number of bases targeted and captured (minimum 3X) for each capture kit is shown as a percentage of the total bases found in each dataset per gene. [file 12920_2014_67_MOESM1_ESM.pdf]

Additional file 1: Table S1: Cardiac genomic regions targeted in the CCDS vs Known Gene datasets

							44MB_V2						50MB_V3						51MB_V4						50MB_V5					
	Gene	Bases in CCDS	Bases in Ensembl	Bases in Known Gene	CCDS within Ensembl	CCDS within Known Gene	CCDS Target	CCDS Capture	Ensembl Target	Ensembl Capture	Known Gene Target	Known Gene Capture	CCDS Target	CCDS Capture	Ensembl Target	Ensembl Capture	Known Gene Target	Known Gene Capture	CCDS Target	CCDS Capture	Ensembl Target	Ensembl Capture	Known Gene Target	Known Gene Capture	CCDS Target	CCDS Capture	Ensembl Target	Ensembl Capture	Known Gene Target	Known Gene Capture
American College of Medical Genetics	ACTA2	1134	1811	3044	62.6%	37.3%	100.0%	100.0%	69.4%	77.5%	41.5%	49.3%	100.0%	100.0%	69.4%	80.2%	41.5%	51.5%	70.2%	100.0%	51.5%	77.6%	35.9%	57.3%	83.2%	100.0%	55.5%	81.7%	33.0%	52.7%
	ACTC1	1134	4113	3700	27.6%	30.6%	100.0%	100.0%	30.1%	33.0%	33.5%	36.7%	100.0%	100.0%	30.1%	33.3%	33.5%	37.1%	69.1%	100.0%	23.1%	33.6%	25.7%	37.3%	61.2%	100.0%	17.4%	31.0%	19.3%	33.5%
	APOB	13692	14150	14150	96.8%	96.8%	99.5%	99.4%	96.8%	97.5%	96.8%	97.4%	99.6%	99.4%	97.6%	97.6%	97.6%	97.6%	94.4%	99.4%	93.1%	99.1%	93.1%	99.0%	96.7%	100.0%	93.9%	98.9%	93.9%	98.6%
	COL3A1	4401	5600	5541	78.6%	79.4%	100.0%	90.1%	80.8%	62.7%	81.6%	77.2%	100.0%	97.3%	80.8%	66.9%	81.6%	83.5%	86.0%	99.6%	72.7%	70.0%	73.5%	87.3%	93.0%	100.0%	75.3%	67.7%	76.0%	84.9%
	DSC2	2742	5140	5300	53.3%	51.7%	100.0%	97.5%	53.9%	53.9%	53.1%	53.1%	100.0%	97.5%	53.9%	54.6%	53.1%	53.9%	90.2%	100.0%	52.4%	61.7%	51.5%	61.4%	92.1%	100.0%	50.4%	58.3%	49.7%	57.6%
	DSG2	3357	5846	5668	57.4%	59.2%	100.0%	98.7%	58.2%	59.6%	60.0%	61.4%	100.0%	99.4%	58.2%	60.0%	60.0%	61.9%	93.4%	100.0%	55.6%	63.9%	57.3%	65.9%	97.2%	100.0%	56.6%	64.5%	58.4%	66.1%
	DSP	8616	9820	9754	87.7%	88.3%	99.4%	98.6%	88.1%	88.9%	88.7%	89.4%	99.4%	99.9%	88.1%	91.2%	88.7%	91.8%	94.3%	100.0%	85.1%	93.3%	85.7%	93.7%	97.5%	100.0%	86.0%	91.5%	86.6%	91.9%
	FBN1	8616	11822	11824	72.9%	72.9%	99.6%	100.0%	73.8%	75.1%	73.8%	75.1%	99.6%	100.0%	73.8%	75.9%	74.4%	76.4%	87.9%	100.0%	64.7%	75.9%	65.2%	76.4%	95.9%	100.0%	70.4%	75.1%	70.9%	75.4%
	GLA	1290	1325	1927	97.4%	66.9%	100.0%	100.0%	100.0%	100.0%	72.6%	82.5%	100.0%	100.0%	100.0%	100.0%	72.6%	84.2%	80.1%	100.0%	78.7%	100.0%	62.8%	88.0%	92.2%	100.0%	90.1%	100.0%	66.2%	85.0%
	KCNH2	3857	5953	6620	64.8%	58.3%	92.0%	64.4%	60.3%	36.5%	55.5%	39.2%	92.0%	76.8%	60.3%	45.5%	55.5%	50.0%	87.1%	100.0%	59.1%	61.4%	58.3%	72.4%	85.9%	98.8%	53.5%	57.8%	52.9%	68.8%
	KCNQ1	2031	3541	3635	57.4%	55.9%	95.3%	71.1%	62.4%	43.8%	60.8%	46.3%	95.3%	94.5%	62.4%	55.0%	63.4%	63.7%	92.3%	98.3%	60.7%	57.2%	61.3%	67.8%	91.8%	98.0%	55.6%	58.6%	56.6%	68.0%
	LDLR	2583	5355	5685	48.2%	45.4%	100.0%	98.3%	48.1%	50.3%	48.6%	49.8%	100.0%	100.0%	48.1%	50.9%	48.6%	52.7%	87.8%	100.0%	46.6%	56.3%	45.4%	57.5%	91.9%	100.0%	52.9%	60.5%	51.3%	61.1%
	LMNA	2106	3799	5690	55.4%	37.0%	95.7%	74.5%	54.8%	34.7%	46.9%	35.9%	95.7%	90.5%	54.8%	43.3%	48.0%	52.8%	86.4%	100.0%	67.5%	53.6%	58.7%	83.1%	91.6%	97.6%	57.9%	49.2%	50.7%	66.8%
	MYBPC3	3825	4255	4305	89.9%	88.9%	96.0%	63.7%	88.9%	61.1%	87.9%	58.3%	100.0%	99.8%	92.5%	92.5%	91.4%	92.5%	91.6%	99.8%	85.4%	95.0%	84.4%	94.0%	93.4%	100.0%	86.6%	94.4%	85.6%	94.5%
	MYH11	5971	6929	12777	86.2%	46.7%	98.8%	99.1%	86.7%	89.5%	49.1%	54.3%	98.8%	100.0%	86.7%	90.4%	49.1%	56.8%	81.4%	100.0%	73.6%	94.3%	44.9%	62.0%	90.1%	100.0%	79.4%	92.9%	47.5%	58.6%
	MYH7	5808	6127	6070	94.8%	95.7%	99.4%	100.0%	96.3%	97.4%	97.2%	98.4%	99.4%	100.0%	96.3%	97.4%	97.2%	98.4%	65.4%	97.9%	64.2%	95.4%	64.8%	96.3%	67.0%	99.6%	65.2%	97.3%	65.8%	98.0%
	MYL2	501	814	844	61.5%	59.4%	100.0%	100.0%	69.7%	83.4%	69.3%	83.8%	100.0%	100.0%	69.7%	89.3%	69.3%	89.7%	99.4%	100.0%	75.6%	97.8%	76.5%	98.0%	100.0%	100.0%	75.9%	96.3%	76.9%	96.2%
	MYL3	588	931	936	63.2%	62.8%	100.0%	100.0%	71.1%	74.8%	70.7%	74.3%	100.0%	100.0%	71.1%	79.6%	70.7%	79.0%	77.9%	100.0%	61.1%	84.3%	60.8%	83.8%	87.8%	100.0%	65.2%	81.7%	64.9%	80.4%
	MYLK	5745	15195	10091	37.8%	56.9%	100.0%	97.1%	38.8%	6.1%	58.9%	61.0%	100.0%	97.4%	38.8%	6.3%	58.9%	62.5%	82.4%	100.0%	32.3%	6.5%	50.7%	67.3%	78.6%	100.0%	30.2%	5.8%	46.8%	65.0%
	PCSK9	2079	3912	4168	53.1%	49.9%	97.9%	67.1%	54.9%	14.2%	51.5%	33.9%	97.9%	91.1%	54.9%	21.3%	51.5%	49.6%	80.0%	100.0%	49.7%	28.3%	46.6%	64.8%	85.1%	92.8%	47.9%	28.3%	44.9%	56.8%
	PKP2	2646	4315	4453	61.3%	59.4%	96.3%	94.7%	59.4%	58.9%	58.3%	59.9%	96.3%	98.7%	59.4%	62.1%	58.3%	63.1%	71.2%	94.6%	45.6%	59.0%	44.9%	60.1%	67.6%	95.7%	42.6%	59.7%	42.3%	60.7%
	PRKAG2	1710	3901	8158	43.8%	21.0%	100.0%	99.5%	46.6%	37.2%	24.1%	30.1%	100.0%	100.0%	49.3%	44.0%	25.4%	36.1%	90.4%	100.0%	57.8%	51.0%	30.9%	45.3%	94.8%	100.0%	52.4%	48.1%	28.5%	42.9%
	RYR2	14904	16774	16603	88.9%	89.8%	99.1%	97.1%	89.3%	87.6%	89.9%	88.3%	100.0%	100.0%	90.1%	90.2%	90.7%	91.8%	91.7%	99.9%	83.9%	90.7%	84.5%	93.2%	95.8%	100.0%	86.8%	90.7%	87.4%	93.0%
	SCN5A	6143	8716	8717	70.5%	70.5%	99.0%	94.8%	71.0%	67.3%	71.1%	69.1%	100.0%	100.0%	71.8%	70.8%	71.8%	73.4%	88.8%	100.0%	64.2%	71.1%	64.1%	73.6%	88.5%	100.0%	62.9%	69.5%	62.9%	71.9%
	SMAD3	1352	6256	6635	21.6%	20.4%	94.5%	93.6%	21.6%	21.8%	20.4%	20.5%	94.5%	94.5%	21.6%	24.0%	20.4%	22.6%	88.5%	100.0%	20.8%	25.9%	20.8%	25.6%	93.0%	100.0%	19.3%	25.9%	19.5%	24.8%
	TGFBR1	1512	6525	6583	23.2%	23.0%	95.3%	93.6%	23.6%	24.6%	23.4%	24.5%	95.3%	93.6%	23.6%	24.8%	23.4%	24.8%	92.6%	100.0%	25.7%	29.6%	25.0%	28.9%	94.4%	100.0%	23.8%	28.8%	25.2%	29.7%
	TGFBR2	1779	4628	4715	38.4%	37.7%	97.6%	94.7%	37.9%	38.0%	38.0%	38.9%	97.6%	100.0%	37.9%	41.6%	38.0%	42.5%	87.6%	100.0%	36.5%	44.6%	37.4%	45.4%	98.0%	100.0%	37.0%	44.8%	38.0%	44.9%
	TMEM43	1203	3353	5106	35.9%	23.6%	100.0%	99.0%	38.4%	37.7%	25.4%	27.2%	100.0%	100.0%	38.4%	42.7%	25.4%	31.1%	91.5%	100.0%	40.4%	49.9%	29.7%	39.3%	90.4%	100.0%	37.3%	46.6%	27.3%	35.3%
	TNNI3	633	848	861	74.6%	73.5%	100.0%	80.1%	84.1%	84.9%	84.1%	85.1%	100.0%	99.5%	84.1%	99.6%	84.1%	99.7%	81.7%	100.0%	71.8%	100.0%	72.2%	100.0%	92.6%	100.0%	81.5%	100.0%	81.8%	100.0%
	TNNT2	897	1423	2472	63.0%	36.3%	100.0%	99.2%	70.9%	72.9%	51.1%	63.8%	100.0%	100.0%	70.9%	81.0%	52.1%	69.8%	91.5%	94.9%	84.5%	78.9%	59.0%	62.7%	97.7%	100.0%	75.6%	91.5%	56.9%	71.8%
	TPM1	1350	3690	3611	36.6%	37.4%	96.5%	80.9%	41.1%	17.9%	42.0%	48.5%	96.5%	80.9%	41.1%	19.1%	42.0%	54.5%	91.5%	100.0%	53.1%	25.3%	52.3%	71.3%	93.3%	100.0%	54.1%	25.3%	53.3%	70.1%
CHD Associated Genes	BRAF	2301	2498	2964	92.1%	77.6%	95.4%	94.0%	91.1%	92.0%	76.8%	82.3%	95.4%	95.9%	91.1%	93.8%	76.8%	84.2%	80.6%	100.0%	79.9%	99.7%	67.6%	89.9%	73.7%	100.0%	68.9%	99.7%	58.1%	83.7%
	CFC1	672	1052	1781	63.9%	37.7%	100.0%	74.3%	71.0%	77.6%	41.9%	44.8%	100.0%	92.9%	71.0%	92.4%	41.9%	54.5%	0.0%	0.0%	0.0%	0.0%	0.0%	0.0%	95.7%	86.6%	68.4%	88.9%	40.4%	50.4%
	CHD7	8994	11883	13016	75.7%	69.1%	96.1%	98.7%	73.5%	29.8%	67.1%	70.3%	100.0%	100.0%	77.0%	30.4%	70.3%	73.8%	91.6%	100.0%	72.4%	30.8%	66.1%	75.5%	94.3%	100.0%	72.1%	30.2%	65.8%	74.2%
	ELN	2208	4019	3538	54.9%	62.4%	95.9%	93.8%	58.7%	52.5%	63.0%	63.8%	95.9%	99.1%	59.7%	56.5%	63.0%	70.0%	82.2%	100.0%	57.4%	57.7%	58.0%	75.7%	86.5%	100.0%	57.7%	57.9%	60.4%	76.9%
	GATA4	1329	3799	3914	35.0%	34.0%	97.6%	57.3%	35.3%	22.8%	34.2%	23.4%	97.6%	64.9%	35.3%	23.2%	34.2%	28.7%	86.2%	100.0%	35.3%	25.5%	34.2%	46.4%	91.8%	92.3%	35.5%	23.8%	34.4%	40.4%
	HRAS	633	1239	1240	51.1%	51.0%	100.0%	96.5%	58.2%	51.7%	58.1%	54.8%	100.0%	100.0%	58.2%	59.5%	58.1%	66.0%	92.7%	100.0%	62.2%	73.9%	62.2%	80.6%	100.0%	100.0%	53.9%	65.5%	53.9%	71.5%
	JAG1	3657	6319	7067	57.9%	51.7%	100.0%	97.8%	58.5%	51.7%	52.4%	54.2%	100.0%	97.9%	58.5%	51.7%	52.4%	55.3%	88.3%	100.0%	55.1%	51.7%	50.7%	62.6%	94.5%	100.0%	55.1%	51.7%	50.1%	60.6%
	PTPN11	1786	6416	6593	27.8%	27.1%	97.8%	99.2%	30.0%	22.2%	29.2%	28.1%	97.8%	99.2%	30.0%	23.0%	29.2%	29.0%	22.7%	81.0%	11.2%	27.1%	10.9%	30.3%	33.8%	100.0%	11.5%	25.7%	11.2%	30.8%
	RAF1	1947	3317	3292	58.7%	59.1%	100.0%	100.0%	61.1%	63.8%	61.6%	64.2%	100.0%	100.0%	61.1%	65.2%	61.6%	65.7%	81.9%	100.0%	52.7%	66.6%	53.1%	67.1%	90.9%	100.0%	54.5%	63.6%	54.9%	63.8%
	SOS1	4002	8541	11842	46.9%	33.8%	100.0%	100.0%	47.9%	50.0%	35.3%	39.2%	100.0%	100.0%	47.9%	50.0%	35.3%	39.3%	94.5%	100.0%	46.1%	50.1%	34.5%	39.3%	95.7%	100.0%	45.6%	49.9%	33.8%	38.6%
	TBX1	1786	2821	2816	63.3%	63.4%	73.3%	66.8%	52.6%	29.4%	52.7%	50.5%	73.3%	78.3%	52.6%	35.5%	52.7%	63.5%	83.0%	89.9%	61.6%	39.0%	61.7%	74.6%	79.8%	94.0%	54.6%	43.4%	54.7%	74.5%
	TBX5	1557	3902	4440	39.9%	35.1%	100.0%	99.0%	43.2%	26.9%	37.9%	38.8%	100.0%	100.0%	43.2%	27.1%	37.9%	40.5%	84.5%	100.0%	42.1%	27.1%	37.0%	46.3%	94.7%	100.0%	41.2%	27.1%	36.2%	41.5%
	ZIC3	1404	3472	3942	40.4%	35.6%	82.8%	69.8%	36.0%	33.8%	31.7%	29.5%	88.8%	100.0%	38.4%	48.9%	33.8%	43.0%	96.9%	100.0%	44.3%	54.4%	39.0%	47.9%	99.6%	100.0%	41.4%	55.0%	36.5%	47.1%
	KRAS	687	7179	6830	9.6%	10.1%	100.0%	100.0%	8.9%	1.7%	11.2%	13.2%	100.0%	100.0%	8.9%	1.7%	11.2%	13.3%	75.1%	100.0%	6.0%	2.6%	7.8%	13.0%	94.2%	100.0%	11.8%	6.6%	14.0%	21.6%
	MAP2K1	1182	3421	2664	34.6%	44.4%	100.0%	93.2%	35.5%	36.6%	45.6%	46.9%	100.0%	100.0%	35.5%	40.7%	45.6%	52.2%	72.1%	100.0%	39.0%	67.9%	44.4%	69.3%	63.7%	100.0%	33.9%	61.5%	36.9%	64.8%
	NF1	8581	13496	18427	63.6%	46.6%	97.4%	99.3%	62.8%	14.0%	46.7%	51.3%	97.4%	99.3%	63.1%	14.1%	47.2%	52.5%	70.6%	83.8%	48.7%	15.2%	36.8%	47.7%	78.5%	95.0%	52.4%	15.2%	39.6%	52.3%
	NOTCH1	7668	9405	10035	81.5%	76.4%	100.0%	64.9%	82.6%	54.0%	78.1%	50.7%	100.0%	91.8%	82.6%	76.6%	78.1%	73.5%	92.9%	99.6%	77.4%	83.6%	75.3%	83.1%	95.8%	100.0%	78.6%	83.8%	76.8%	83.7%
	NKX2-5	1102	1711	1963	64.4%	56.1%	78.9%	80.5%	52.5%	62.9%	47.7%	52.9%	78.9%	88.5%	52.5%	73.0%	47.7%	69.4%	99.2%	100.0%	65.3%	79.1%	69.7%	83.8%	95.4%	100.0%	56.9%	77.7%	64.1%	81.6%
	NRAS	570	4456	4461	12.8%	12.8%	100.0%	100.0%	13.1%	13.4%	13.0%	13.3%	100.0%	100.0%	13.1%	13.4%	13.0%	13.3%	94.6%	100.0%	12.6%	13.4%	12.6%	13.3%	100.0%	100.0%	13.4%	13.4%	13.3%	13.3%
TOTAL:		166271	275813	300468	60.3%	98.0%	98.0%	92.5%	60.8%	50.0%	56.5%	55.7%	98.5%	97.5%	61.2%	53.5%	57.0%	60.4%	85.9%	98.1%	56.0%	55.9%	52.7%	64.4%	89.8%	99.3%	56.4%	55.8%	53.0%	63.6%
